# Multivessel Versus Single-Vessel Coronary Endarterectomy as an Adjunct to Coronary Artery Bypass Grafting: A Meta-Analysis of Short-Term Outcomes and Implications for Target-Vessel Selection

**DOI:** 10.1093/icvts/ivag238

**Published:** 2026-08-26

**Authors:** Joshua J Hon, Arian Arjomandi Rad, Abdullah Tahir, Elisa Salagean, Marco Moscarelli, Dimitrios E Magouliotis, Roberto Casula, Thanos Athanasiou

**Affiliations:** Department of Surgery and Cancer, Imperial College London, London, W12 0NN, United Kingdom; Department of Cardiothoracic Surgery, Oxford University Hospitals NHS Foundation Trust, Oxford, OX3 9DU, United Kingdom; Department of Trauma and Orthopaedics, University Hospitals of North Midlands, ST4 6QG, United Kingdom; Department of Cardiothoracic Surgery, Oxford University Hospitals NHS Foundation Trust, Oxford, OX3 9DU, United Kingdom; Department of Surgery and Cancer, Imperial College London, London, W12 0NN, United Kingdom; Department of Cardiovascular Surgery, Maria Eleonora Hospital, GVM Care & Research, Palermo, 90135, Italy; Department of Cardiac Surgery Research, Lankenau Institute for Medical Research, Main Line Health, Wynnewood, PA 19096, United States; Department of Cardiothoracic Surgery, Hammersmith Hospital Imperial College Healthcare NHS Trust, London, W12 0HS, United Kingdom; Department of Surgery and Cancer, Imperial College London, London, W12 0NN, United Kingdom; Department of Cardiothoracic Surgery, Hammersmith Hospital Imperial College Healthcare NHS Trust, London, W12 0HS, United Kingdom

**Keywords:** coronary endarterectomy, coronary artery bypass grafting, diffuse coronary artery disease, meta-analysis, perioperative outcomes, target-vessel selection

## Abstract

**Objectives:**

Coronary endarterectomy (CE) extends complete revascularization to patients with diffuse coronary disease unsuitable for conventional bypass but adds perioperative risk to coronary artery bypass grafting (CABG). Whether the number of vessels endarterectomized influences early outcomes has not been formally synthesized. We compared short-term outcomes of single-vessel and multivessel CE-CABG.

**Methods:**

MEDLINE, Embase, and CENTRAL were searched for studies of CE-CABG reporting outcomes stratified by single-vessel vs multivessel CE within the same cohort. The primary outcome was 30-day or in-hospital mortality. Risk ratios (RR) were pooled using DerSimonian–Laird random-effects models. Sensitivity analyses included leave-one-out analysis, Hartung–Knapp–Sidik–Jonkman (HKSJ) adjustment, and univariate meta-regression on study-level covariates. Risk of bias was assessed with ROBINS-I.

**Results:**

Nine studies (1988-2022; 7302 patients; 4731 single-vessel CE, 2571 multivessel CE) met inclusion criteria. Multivessel CE was associated with higher mortality (RR, 2.59; 95% CI, 1.78-3.77), higher perioperative myocardial infarction (RR, 2.09; 95% CI, 1.67-2.62), and higher stroke (RR, 1.62; 95% CI, 1.01-2.61). Intra-aortic balloon pump use did not differ. Findings were stable on leave-one-out, HKSJ adjustment, and exclusion of pre-2000 studies. No single covariate accounted for the multivessel effect on meta-regression.

**Conclusions:**

Multivessel CE substantially increases early mortality and infarction relative to single-vessel CE. The signal is consistent across analyses but the evidence is largely retrospective and unadjusted. These data support a selective approach to CE in which the prognostic value of each target vessel is weighed against the cumulative operative cost, rather than routine extension of endarterectomy to multiple territories.

## GRAPHICAL ABSTRACT

**Figure:**
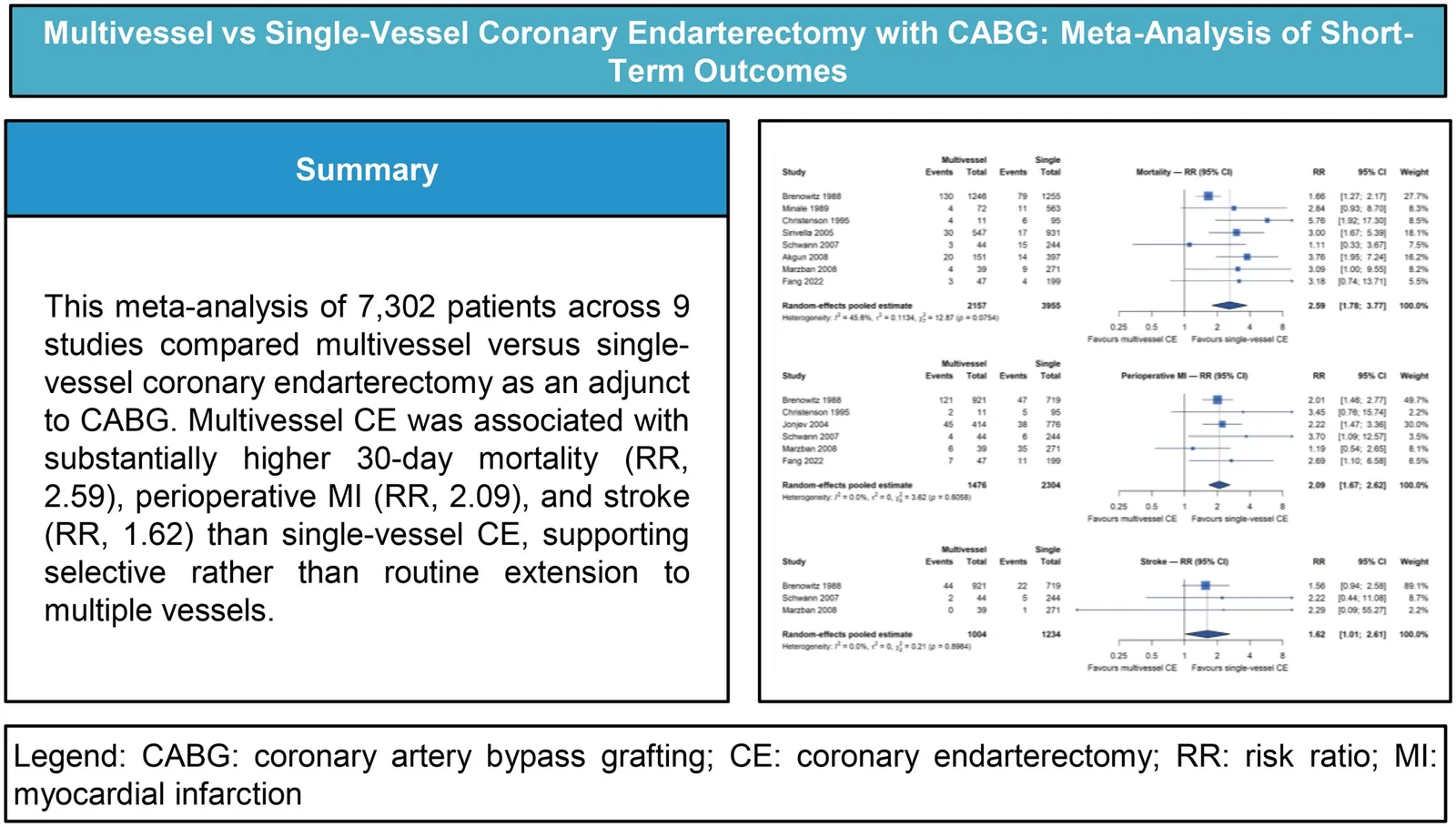

## INTRODUCTION

Coronary endarterectomy (CE) restores antegrade flow in arteries that cannot be grafted by conventional means. It remains the surgical option of last resort for diffusely diseased coronary arteries, particularly in patients in whom percutaneous intervention has been exhausted or judged futile. A previous meta-analysis of more than 100 000 patients confirmed that adding CE to coronary artery bypass grafting (CABG) raises early mortality and morbidity compared with isolated CABG, although baseline disease severity accounts for some of the apparent excess.^1^

Within the heterogeneous CE-CABG population, the number of arteries endarterectomized varies widely. Some patients undergo a single CE, typically of the left anterior descending (LAD) or right coronary artery (RCA); others have 2, 3, or more vessels endarterectomized in the same operation. Single-centre series have repeatedly suggested that operating on more than one vessel carries worse perioperative results, but the literature has not been systematically pooled. The implicit question is whether considering CE should be approached as a routine extension to bypass whenever an ungraftable target is encountered, or as a targeted adjunct deployed selectively when the prognostic value of the territory justifies the potential procedural cost.

We undertook a focused meta-analysis of studies reporting outcomes stratified by the number of vessels endarterectomized. The objective was to estimate the relative early risk of multivessel vs single-vessel CE-CABG, to explore whether the effect is explained by measured baseline characteristics, and to inform a framework for target vessel selection in patients with diffuse coronary disease.

## METHODS

The study was conducted in accordance with the Preferred Reporting Items for Systematic Review and Meta-Analyses (PRISMA) 2020 statement,^2^ and registered on the International Prospective Register of Systematic Review (PROSPERO; CRD420261395957).

### Search strategy

MEDLINE (via PubMed), Embase (via Ovid), and the Cochrane Central Register of Controlled Trials (CENTRAL) (via the Cochrane Library) were searched from inception to 14 May 2026. Search strings combined free-text terms for “coronary endarterectomy” and “coronary artery bypass grafting,” and excluded terms relating to carotid, brachiocephalic, and aortoiliac procedures. The complete search strategy is reported in **Table S1**.

### Eligibility criteria

Eligible studies enrolled adults undergoing CE as an adjunct to CABG and reported short-term outcomes separately for patients receiving single-vessel vs multivessel CE within the same cohort. Multivessel CE was defined as endarterectomy of 2 or more coronary arteries during the index operation. The minimum acceptable sample size was 20 CE patients. Case reports, paediatric studies, studies in which CE could not be distinguished from carotid endarterectomy, and conference abstracts without full-text availability were excluded. Studies were restricted to those reporting in English.

### Study selection and data extraction

Two reviewers independently screened titles, abstracts, and full-texts. Disagreements were resolved by consensus. Data were extracted using a standardized sheet capturing study characteristics, baseline population variables, operative details, and outcomes. Where event counts for one arm were not directly reported but could be derived from totals and the complementary arm, this was performed.

### Outcomes

The primary outcome was 30-day or in-hospital mortality. Secondary outcomes were perioperative myocardial infarction (MI), stroke, postoperative renal failure, intra-aortic balloon pump (IABP) use, reoperation for bleeding, postoperative atrial fibrillation, and intensive care unit length of stay.

### Risk of bias assessment

Risk of bias was appraised using the Risk Of Bias In Non-randomized Studies–of Interventions (ROBINS-I) tool,^3^ with overall judgement assigned according to the highest-risk domain.

### Statistical analysis

Risk ratios (RRs) with 95% confidence intervals were calculated for dichotomous outcomes and pooled using random-effects meta-analysis with the DerSimonian-Laird estimator. The Mantel–Haenszel method was used for pooling, with a continuity correction of 0.5 applied to studies containing a zero-event arm. Heterogeneity was quantified with *I*^2^ and Cochran’s *Q*. Prespecified sensitivity analyses for the primary outcome included leave-one-out analysis, exclusion of studies from which event counts were derived rather than directly reported, restriction to studies published from the year 2000 onwards, exclusion of the largest-weight study, and Hartung–Knapp–Sidik–Jonkman (HKSJ) adjustment of the random-effects model to provide more conservative confidence intervals in the small study number setting. For perioperative MI, a sensitivity analysis excluding the non-randomized post hoc subgroup^4^ was also conducted. HKSJ-adjusted estimates were also computed for the secondary outcomes.

To explore the contribution of baseline confounders to the observed effects, univariate random-effects meta-regression was performed for mortality on 5 study-level covariates: prevalence of triple-vessel disease, mean left ventricular ejection fraction (LVEF), prevalence of diabetes mellitus, proportion of cases performed on cardiopulmonary bypass, and publication year. Publication bias was assessed using funnel plot inspection and Egger’s regression test for the primary outcome. Outcomes reported by fewer than 3 studies were summarized narratively.

All analyses were performed in R version 4.5.2 (R Foundation for Statistical Computing, Vienna, Austria) with the meta package.

## RESULTS

### Study selection and characteristics

The literature search identified 1505 records. Following removal of 370 duplicates, 1135 records were screened at the title and abstract level, of which 139 full-text reports were assessed for eligibility. A further 130 reports were excluded at full-text, leaving 9 studies that met all inclusion criteria. The PRISMA 2020 flow diagram is presented in **Figure 1**.

**Figure 1.**
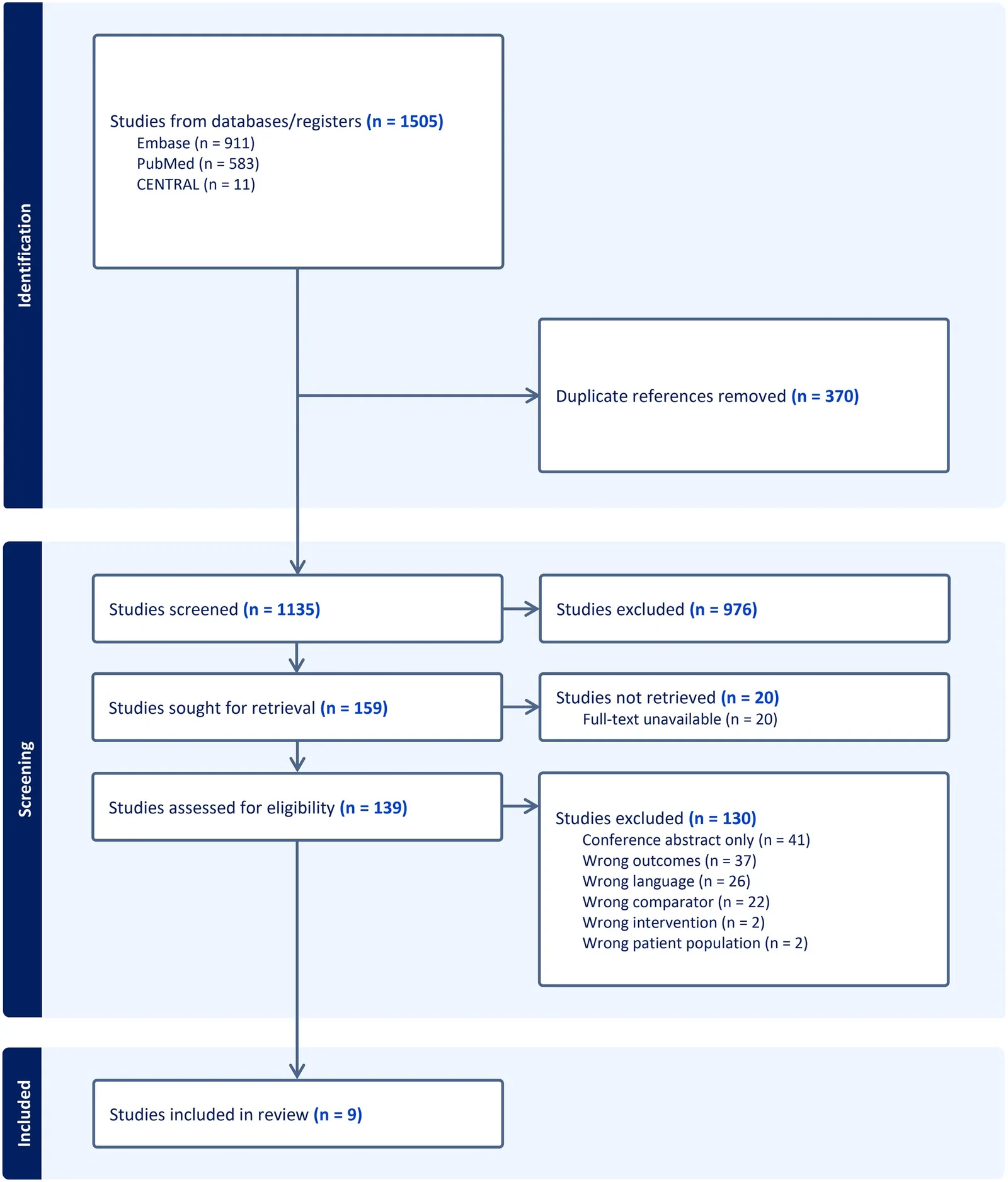
PRISMA 2020 Flow Diagram of Study Selection

The 9 included studies were published between 1988 and 2022 and contributed data on 7302 patients (4731 single-vessel CE; 2571 multivessel CE) (**Table 1**). Eight were retrospective cohorts,^5–12^ and one was a randomized trial of intraoperative prostacyclin in which the single-vessel vs multivessel comparison was a post hoc subgroup analysis.^4^ Multivessel CE was defined as 2 or more endarterectomized vessels in all studies; the proportion of patients in the multivessel arm ranged from 10% to 50%.

**Table 1.** Characteristics of the 9 Included Studies

Study	Country	Design	Study period	On/off-pump	Total CE (*n*)	Single CE (*n*)	Multi CE (*n*)	Definition of multi
Brenowitz 1988	United States	Retrospective cohort	1978-1986	On-pump	2501	1255	1246	≥2 vessels (Group C)
Minale 1989	West Germany	Retrospective cohort	1980-1987	On-pump	635	563	72	≥2 vessels
Christenson 1995	Switzerland	Retrospective cohort	1990-1994	On-pump	106	95	11	≥2 vessels (LAD + RCA in 9, LAD + Cx in 2)
Jonjev 2004	Yugoslavia (Serbia)	RCT (PGI2 vs control); single vs multi is post hoc	1994-1999	On-pump	1190	776	414	≥2 vessels (double 154 + triple 260)
Sirivella 2005	United States (NJ)	Retrospective cohort	1985-2002	Both (52 off-pump from 2000)	1478	931	547	≥2 vessels (37% multi; 12% triple-vessel)
Schwann 2007	United States (Ohio)	Retrospective cohort	1996-2003	Both (on 283/off 5)	288	244	44	≥2 vessels (1.18 CEs/patient)
Akgun 2008	Turkey	Retrospective cohort	1996-2004	On-pump (off in selected)	548	397	151	≥2 vessels
Marzban 2008	Iran	Retrospective cross-sectional	January 2002-March 2006	On-pump	310	271	39	2 vessels (double)
Fang 2022	China	Retrospective cohort	January 2012-December 2014	Off-pump	246	199	47	≥2 vessels (LAD/RCA/LCx)

### Primary outcome: mortality

Multivessel CE was associated with higher 30-day or in-hospital mortality (8 studies; 9.2% vs 3.9%; RR, 2.59; 95% CI, 1.78-3.77; *P* < .001; *I*^2^ = 46%; **Figure 2A**). The HKSJ-adjusted estimate was concordant (RR, 2.59; 95% CI, 1.75-3.85; *P* < .001).

**Figure 2.**
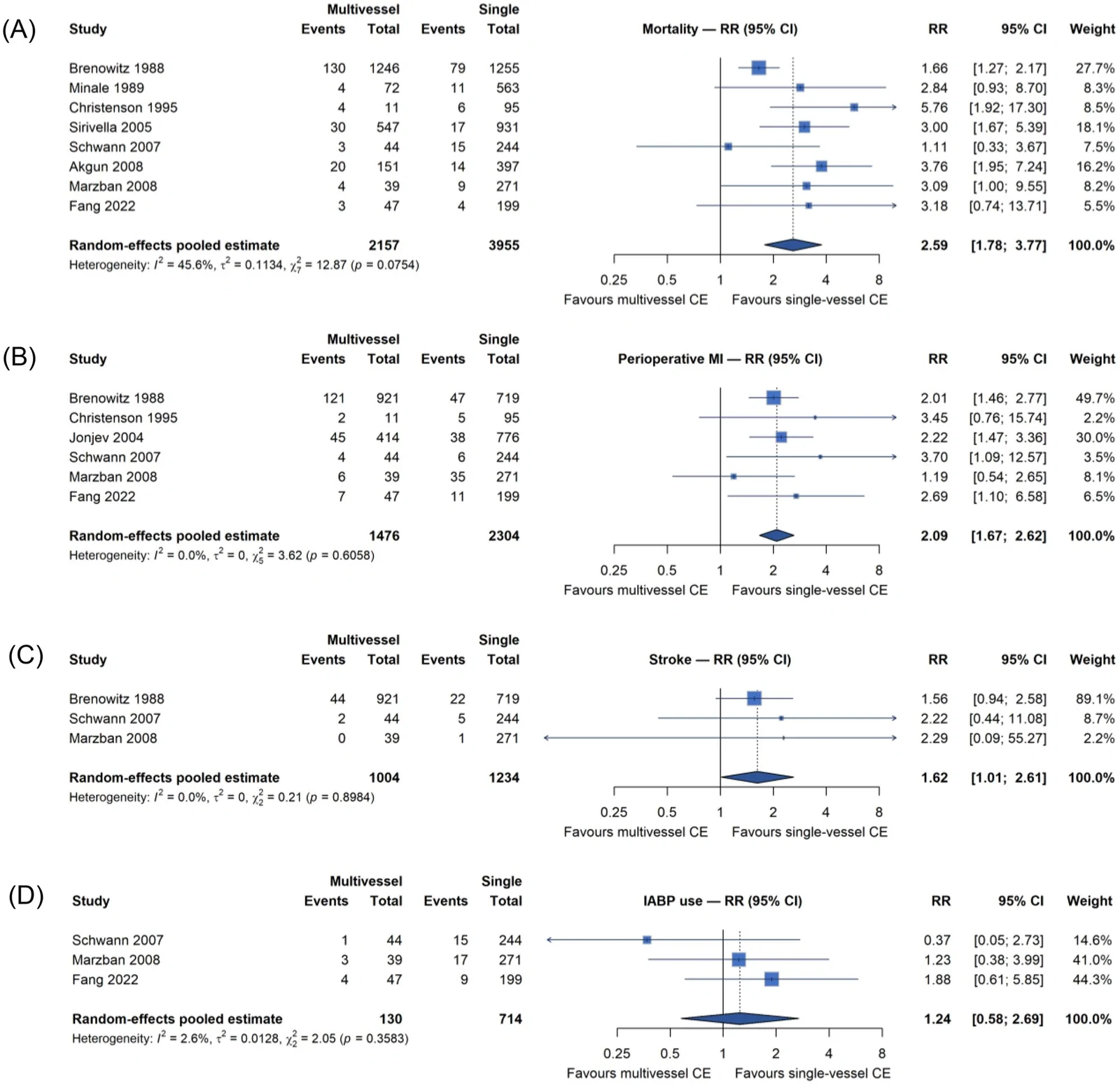
Forest Plots of Pooled RRs Comparing Multivessel With Single-Vessel CE. (A) 30-Day or in-hospital mortality; (B) Perioperative MI; (C) Stroke; (D) IABP use

Sensitivity analyses confirmed the direction and magnitude of the effect (**Table 2**). Leave-one-out analysis produced pooled RRs between 2.37 and 3.13. Exclusion of the largest-weight study^12^ increased the pooled RR to 3.13 (95% CI, 2.24-4.39) with heterogeneity falling to 0. Restricting to studies published in 2000 or later yielded an RR of 2.95 (95% CI, 2.03-4.28); restricting to studies with directly reported event counts yielded an RR of 2.88 (95% CI, 1.65-5.02).

**Table 2.** Sensitivity Analyses for the Primary Outcome of 30-Day or in-Hospital Mortality

Analysis	Studies	RR	95% CI	*I* ^2^
Primary (DerSimonian-Laird)	8	2.59	1.78-3.77	46%
HKSJ-adjusted	8	2.59	1.75-3.85	46%
Excluding derived event counts	5	2.88	1.65-5.02	60%
Excluding pre-2000 studies	5	2.95	2.03-4.28	0%
Excluding Brenowitz 1988 (largest weight)	7	3.13	2.24-4.39	0%
Leave-one-out	8	2.37-3.13	–	–

### Meta-regression and confounding by indication

No single study-level covariate significantly modified the mortality effect (**Table 3**). Publication year and era explained the largest proportion of between-study variance (*R*^2^ = 66% and 31%, respectively) but neither reached statistical significance (*P* = .15 and *P* = .47). The 4 biological covariates (triple-vessel disease prevalence, mean LVEF, diabetes prevalence, and on-pump proportion) produced null findings with *R*^2^ of zero. The mortality elevation associated with multivessel CE was, therefore, not reducible to any single measured baseline characteristic. Bubble plots are presented in **Figure S1**.

**Table 3.** Univariate Meta-Regression for Mortality on Study-Level Covariates

Covariate	Studies	RR per unit	95% CI	*P*-value	*R* ^2^
Triple-vessel disease (%)	5	1.001	0.982-1.019	.95	0%
Mean LVEF (%)	5	1.022	0.933-1.118	.64	0%
Diabetes mellitus (%)	7	0.995	0.963-1.028	.75	0%
On-pump (%)	8	0.998	0.981-1.015	.81	0%
Publication year	8	1.022/year	0.992-1.052	.15	66%
Era (post-2000 vs pre-2000)	8	1.287	0.650-2.551	.47	31%

### Secondary outcomes

Perioperative MI was reported with vessel-number stratification by 6 studies. The pooled rate was 12.5% following multivessel CE compared to 6.2% following single-vessel CE (RR, 2.09; 95% CI, 1.67-2.62; *P* < .001; *I*^2^ = 0%; **Figure 2B**). The HKSJ-adjusted estimate was 2.09 (95% CI, 1.62-2.69; *P* < .001). MI definitions varied across studies, ranging from electrocardiographic criteria alone to multi-marker definitions incorporating troponin and wall-motion abnormalities. Jonjev et al. the only randomized trial and the sole study in which the vessel-number comparison derived from a post hoc non-randomized subgroup, contributed to this outcome but not to mortality. Excluding it left the estimate essentially unchanged (RR, 2.04; 95% CI, 1.55-2.67; *I*^2^ = 0%).

Stroke was reported by 3 studies. Multivessel CE was associated with a higher pooled risk (4.6% vs 2.3%; RR, 1.62; 95% CI, 1.01-2.61; *P* = .045; *I*^2^ = 0%; **Figure 2C**). The HKSJ-adjusted estimate was 1.62 (RR, 1.16-2.28; *P* = .026). The result was primarily driven by Brenowitz et al.,^12^ the largest contributor.

IABP use was reported by 3 studies and did not differ between groups (6.2% vs 5.7%; RR, 1.24; 95% CI, 0.58-2.69; *P* = .58; *I*^2^ = 3%; **Figure 2D**). Postoperative atrial fibrillation, reoperation for bleeding, and renal failure were each reported by 2 or fewer studies and were not pooled. Where reported, rates were numerically higher after multivessel CE without reaching statistical significance.

We examined whether outcomes could be separated for double- vs triple-vessel CE to permit a dose-response analysis. Only one study^4^ reported these cases separately, and only for perioperative MI; a formal dose-response synthesis was therefore not feasible.

### Adjusted estimates

Adjusted estimates comparing multivessel with single-vessel CE were available from 2 studies. Fang et al. reported an adjusted odds ratio of 9.04 (95% CI, 2.20-37.19; *P* = .002) for perioperative MI in patients undergoing multivessel CE compared with isolated LAD endarterectomy.^5^ Christenson et al. identified 2-vessel CE as an independent predictor of in-hospital mortality (*P* = .011) but did not report a point estimate.^11^

### Publication bias and risk of bias

Funnel plot inspection suggested modest asymmetry (**Figure S2**). Egger’s regression test did not reach significance (estimate, 1.39; *P* = .104), although the analysis is underpowered at less than 10 studies. The majority of studies were rated at overall serious risk of bias on ROBINS-I, principally because of unadjusted confounding (**Table S2**).

## DISCUSSION

This systematic review and meta-analysis of 9 studies and 7302 patients shows a substantially higher early hazard for multivessel than single-vessel CE. Mortality was 2.6-fold higher, perioperative MI 2.1-fold higher and stroke 1.6-fold higher. The mortality result was stable across leave-one-out analysis, HKSJ adjustment, exclusion of derived event counts, exclusion of pre-2000 studies, and exclusion of the largest contributing study.

Several mechanisms plausibly contribute. Patients selected for multivessel CE are a sicker subset to begin with: they have more diffuse disease, higher rates of triple-vessel involvement, and a greater burden of unrevascularizable territory. The decision to perform multivessel CE is rarely made on prognostic grounds preoperatively. In practice, it is made intraoperatively, when a second or third target vessel is found to be ungraftable by conventional means; multivessel CE is, therefore, a marker of anatomical necessity rather than a deliberate revascularization strategy. The patients in the multivessel arm of these studies are best understood as those in whom multiple territories were simultaneously unsuitable for bypass, an anatomical phenotype that itself confers risk. Consistent with this, the studies that specify how the decision was reached describe it as an intraoperative judgement made once a target is found ungraftable rather than a preoperative plan, and none report outcomes separately for planned vs unplanned endarterectomy.

Another factor is procedural: each additional endarterectomy extends operative duration, cardiopulmonary bypass time, and aortic cross-clamp time, all of which are independently associated with perioperative mortality and morbidity in cardiac surgery.^13^^,^^14^ The dissection itself exposes a larger area of endothelium that is susceptible to acute thrombosis and the inflammatory cascade triggered by reperfusion, in turn increases postoperative anticoagulation requirements. Multivessel CE, therefore, compounds 2 risk modifiers: patient-level anatomical severity and operation-level physiological burden. Operating and bypass durations were not systematically reported in the included studies and could not be extracted as covariates.

Univariate meta-regression on triple-vessel disease prevalence, LVEF, diabetes prevalence, on-pump proportion, year, and era produced no significant modifier of the mortality effect, although power was limited. This null result supports the interpretation that the observed risk is not attributable to any single measured baseline characteristic. It does not rule out confounding by indication operating through unmeasured or composite variables, which a study-level meta-regression cannot adequately address.

The strongest implication for practice concerns patient selection. The wider literature on coronary revascularization has progressively distinguished anatomical completeness from functional completeness, recognizing that revascularizing every disease segment is not invariably superior to a selective strategy targeting territories with the greatest prognostic value.^15^^,^^16^ The present findings argue that CE belongs within this framework. CE should be conceived as a targeted adjunct deployed when the prognostic benefit of revascularizing a specific territory clearly exceeds the procedural cost, rather than as a routine extension applied whenever an ungraftable artery is encountered.

A functional selection framework may follow these principles. For each candidate vessel, the surgeon should weigh 4 considerations: the volume of viable myocardium supplied and the ischaemic burden; the prognostic importance of the territory, with the LAD warranting separate weight given its established impact on long-term survival; the feasibility and effectiveness of alternatives, including hybrid revascularization with percutaneous coronary intervention and optimized medical therapy^17^^,^^18^; and the cumulative operative cost already incurred by any prior endarterectomies in the same operation. Within this framework, LAD endarterectomy in a patient with a large viable anterior territory may often by justified; extension of CE to a second or third non-LAD territory of uncertain ischaemic burden, by contrast, is not supported by the data. Fang et al. found a roughly 9-fold higher perioperative MI risk with multivessel CE than with isolated LAD CE on multivariable adjustment,^5^ which is consistent with this hierarchy. A distinct question, is whether this LAD-first recommendation extends to the converse clinical situation: a candidate for multivessel CE in whom only a single, prognostically critical territory (typically the LAD) is endarterectomized while other ungraftable territories are left to medical therapy or hybrid revascularization. Our data cannot answer this directly, because no included study reports outcomes according to the anatomical indication for CE as distinct from the treatment delivered. The framework presented is nonetheless consistent with restraint in this setting: if the incremental early hazard of each additional endarterectomy exceeds the prognostic value of the territory it revascularizes, then a deliberately limited, LAD-focused endarterectomy may be a defensible strategy.

The implication is that established surgical risk models, namely the European System for Cardiac Operative Risk Evaluation II (EuroSCORE II),^19^ the Society of Thoracic Surgeons Predicted Risk of Mortality (STS-PROM),^20^ and the SYNergy between percutaneous coronary intervention with TAXus (SYNTAX) and cardiac surgery scores,^21^ capture diffuse coronary disease and number of diseased vessels but do not separately account for the number of vessels endarterectomized. The differential risk identified here is not currently reflected in preoperative risk estimation, which may understate the operative hazard for patients who ultimately undergo multivessel CE. Whether this variable warrants incorporation into existing models, or creation of a CE-specific risk tool, deserves evaluation.

A further source of heterogeneity that calls for comment is postoperative antithrombotic management. The endarterectomized endothelial surface is widely held to predispose to graft thrombosis, and a range of regimens including therapeutic heparin bridging to warfarin, dual antiplatelet therapy (DAPT), and other combinations, have been used across the included studies, with several not specifying the exact protocol used. Across the included studies, the specified regimens ranged from a single postoperative antiplatelet agent, through aspirin combined with low molecular weight dextran, to DAPT, and to heparin-bridged oral anticoagulation continued for 2-6 months, typically alongside aspirin. Only the most recent study additionally employed contemporary DAPT for 1 year with lifelong single-agent continuation. Variability in antithrombotic management is, therefore, both a likely contributor to between-study heterogeneity and an evidence gap. Other evidence reinforces this uncertainty: a small retrospective comparison reported markedly higher endarterectomy-target graft patency with 3 months of aspirin plus warfarin than with DAPT alone,^22^ whereas an older trial of preoperatively initiated warfarin and dipyridamole found it underperformed oral anticoagulation,^23^ and the question is the subject of the ongoing PATH-CARE randomized trial.^24^ Neither the 2021 American College of Cardiology/American Heart Association (ACC/AHA) nor the 2026 European Society of Cardiology/European Society for Cardio-Thoracic Surgery (ESC/EACTS) antithrombotic statements make a CE-specific recommendation.^18^^,^^25^

The technique of endarterectomy, its reconstruction, and the conduit chosen also varied across the included studies (**Table S3**). Two broad techniques were used: a closed (traction) endarterectomy through a short arteriotomy at the anastomotic site, and an open endarterectomy through an extended arteriotomy, most often reserved for the diffusely diseased LAD and closed with a venous or internal mammary onlay patch. Several studies used both selectively, converting from closed to open when the distal plaque could not be cleanly extracted. Within the included studies, reconstruction of the endarterectomized LAD with an internal mammary artery, with or without a supplementary venous patch, was associated with lower mortality and better graft patency than saphenous vein reconstruction, a pattern echoed in the wider literature.^26^^,^^27^ These findings align with a notion that conduit selection appears to influence long-term patency at least as much as the endarterectomy technique itself, in which open reconstruction facilitates the mammary onlay that this advantage depends upon. The number of vessels endarterectomized is thus one of several technical details along which these operations differ; conduit and reconstruction cannot be separated from vessel number at the meta-analytic level, and their presence together as covariates is a likely source of the residual heterogeneity we observed. No included study reported outcomes for endarterectomy of previously stented segments, an increasingly encountered scenario for which the available evidence remains confined to small selected series.

Several limitations deserve attention. Most evidence comes from single-centre retrospective cohorts spanning more than 3 decades, during which CE technique, perioperative care, and antiplatelet protocols have all evolved. Endarterectomy technique varied across studies, as did the choice of conduit to the endarterectomized vessel and the use of cardiopulmonary bypass; only one study reported a purely off-pump experience.^5^ Disentangling the contribution of these technical variables from that of vessel number is not possible with 9 studies, but their variability is a likely source of unmeasured heterogeneity. The definition of multivessel CE was consistent (2 or more vessels), but the case mix within the multivessel arm varied: some series included patients with 3 or more endarterectomies in the same group as those with double CE, which would tend to inflate effect size. Baseline characteristics were rarely reported separately for the single-vessel and multivessel groups, so adjustment for confounding by indication was not possible at the meta-analytic level. One included study was a randomized trial of prostacyclin in which the single-vessel vs multivessel comparison was a non-randomized post hoc subgroup; it contributed only to perioperative MI, and its exclusion did not materially alter that estimate. Perioperative MI definitions varied across studies; reassuringly, between-study heterogeneity for this outcome was 0, suggesting that the size of the effect is comparable across definitions. Sparse reporting precluded pooling of certain secondary outcomes such as renal failure and atrial fibrillation. Finally, the synthesis is confined to short-term outcomes; mid- and long-term survival were not routinely reported stratified by the number of vessels endarterectomized.

Future series of CE-CABG should report outcomes stratified by the number of vessels endarterectomized and provide baseline characteristics and adjusted effect estimates for this comparison. We make a specific recommendation that the number of vessels endarterectomized be captured as a discrete field in established cardiac surgical registries, such as those underpinning the STS and EuroSCORE models, and that a CE-specific risk adjustment be derived and externally validated from multicentre data. This would allow the risk-adjusted analyses that the present literature does not support and would allow quantification for decision support.

## CONCLUSION

In a synthesis of 9 studies of CE-CABG, multivessel CE was associated with approximately doubled early mortality, perioperative MI, and stroke risk relative to single-vessel endarterectomy. The findings were consistent across multiple sensitivity analyses and could not be attributed to any single measured baseline characteristic, although the underlying evidence is largely retrospective and unadjusted. These data support a selective approach to CE in which the procedure is targeted at vessels of clear prognostic value, particularly the LAD, and the cumulative operative cost of additional endarterectomies is weighed against the marginal benefit of revascularizing further territories. Routine extension of CE to multiple ungraftable vessels, as a strategy aimed at anatomical completeness, is not supported by the present evidence. Stratified, prospectively collected outcome data are needed to refine this framework.

## Supplementary Material

ivag238_Supplementary_Data

## Data Availability

All data used can be found on academic literature databases given the nature of systematic reviews and meta-analyses. Aggregated data used for analysis may be requested from the corresponding author.

## ABBREVIATIONS

ACC/AHA: American College of Cardiology/American Heart Association
CABG: coronary artery bypass grafting
CE: coronary endarterectomy
CI: confidence interval
ESC/EACTS: European Society of Cardiology/European Association for Cardio-Thoracic Surgery
EuroSCORE II: European System for Cardiac Operative Risk Evaluation II
HKSJ: Hartung–Knapp–Sidik–Jonkman
IABP: intra-aortic balloon pump
LAD: left anterior descending
LCx: left circumflex
LVEF: left ventricular ejection fraction
MI: myocardial infarction
PRISMA: Preferred Reporting Items for Systematic Reviews and Meta-Analyses
PROSPERO: International Prospective Register of Systematic Reviews
RCA: right coronary artery
ROBINS-I: Risk Of Bias In Non-randomised Studies of Interventions
RR: risk ratio
STS-PROM: Society of Thoracic Surgeons Predicted Risk of Mortality
SYNTAX: SYNergy between Percutaneous Coronary Intervention with TAXus and Cardiac Surgery
