# Supplementary material for: Multivessel Versus Single-Vessel Coronary Endarterectomy as an Adjunct to Coronary Artery Bypass Grafting: A Meta-Analysis of Short-Term Outcomes and Implications for Target-Vessel Selection
Source: Interdiscip Cardiovasc Thorac Surg. 2026 Aug 26;41(11):ivag238. doi: 10.1093/icvts/ivag238 (PMC13730572; doi:10.1093/icvts/ivag238)
Supplement: Supplementary file 1 [file ivag238_supplementary_data.zip › R1.Multiple_CE_Supplement_ICVTS JJH (clean).docx]

**Supplementary Table S1.** Full search strategies for each database.

| **#** | **Terms** |
| --- | --- |
| **MEDLINE (via PubMed)** | |
| 1 | "Endarterectomy"[MeSH] |
| 2 | "coronary endarterectomy"[tiab] |
| 3 | "coronary thromboendarterectomy"[tiab] |
| 4 | "CE-CABG"[tiab] |
| 5 | 1 OR 2 OR 3 OR 4 |
| 6 | "Coronary Artery Bypass"[MeSH] |
| 7 | "coronary artery bypass"[tiab] |
| 8 | "coronary bypass"[tiab] |
| 9 | "CABG"[tiab] |
| 10 | "myocardial revascularization"[tiab] |
| 11 | "myocardial revascularisation"[tiab] |
| 12 | 6 OR 7 OR 8 OR 9 OR 10 OR 11 |
| 13 | 5 AND 12 |
| 14 | "Endarterectomy, Carotid"[MeSH] |
| 15 | "carotid endarterectomy"[tiab] |
| 16 | "brachiocephalic"[tiab] |
| 17 | "aortoiliac"[tiab] |
| 18 | 14 OR 15 OR 16 OR 17 |
| 19 | 13 NOT 18 |
| **Embase (via Ovid)** | |
| 1 | endarterectomy/ |
| 2 | coronary endarterectomy.ti,ab. |
| 3 | coronary thromboendarterectomy.ti,ab. |
| 4 | CE-CABG.ti,ab. |
| 5 | 1 OR 2 OR 3 OR 4 |
| 6 | coronary artery bypass graft/ |
| 7 | coronary artery bypass surgery/ |
| 8 | coronary artery bypass grafting.ti,ab. |
| 9 | coronary bypass.ti,ab. |
| 10 | CABG.ti,ab. |
| 11 | myocardial revascularization.ti,ab. |
| 12 | myocardial revascularisation.ti,ab. |
| 13 | 6 OR 7 OR 8 OR 9 OR 10 OR 11 OR 12 |
| 14 | 5 AND 13 |
| 15 | carotid endarterectomy/ |
| 16 | carotid.ti,ab. |
| 17 | brachiocephalic.ti,ab. |
| 18 | aortoiliac.ti,ab. |
| 19 | 15 OR 16 OR 17 OR 18 |
| 20 | 14 NOT 19 |
| **CENTRAL (via Cochrane Library)** | |
| 1 | "coronary endarterectomy":ti,ab,kw |
| 2 | "coronary thromboendarterectomy":ti,ab,kw |
| 3 | "CE-CABG":ti,ab,kw |
| 4 | 1 OR 2 OR 3 |
| 5 | "coronary artery bypass":ti,ab,kw |
| 6 | "coronary bypass":ti,ab,kw |
| 7 | "CABG":ti,ab,kw |
| 8 | "myocardial revascularization":ti,ab,kw |
| 9 | "myocardial revascularisation":ti,ab,kw |
| 10 | 5 OR 6 OR 7 OR 8 OR 9 |
| 11 | 4 AND 10 |
| 12 | "carotid":ti,ab,kw |
| 13 | "brachiocephalic":ti,ab,kw |
| 14 | "aortoiliac":ti,ab,kw |
| 15 | 12 OR 13 OR 14 |
| 16 | 11 NOT 15 |

**Supplementary Table S2.** Risk of bias assessment using the ROBINS-I tool.

| **Study** | **D1: Confounding** | **D2: Selection of participants** | **D3: Classification of intervention** | **D4: Deviations from intended intervention** | **D5: Missing data** | **D6: Measurement of outcome** | **D7: Selection of reported result** | **Overall** |
| --- | --- | --- | --- | --- | --- | --- | --- | --- |
| Brenowitz 1988 | Serious | Moderate | Low | Low | Moderate | Moderate | Moderate | Serious |
| Minale 1989 | Serious | Moderate | Low | Low | Low | Moderate | Moderate | Serious |
| Christenson 1995 | Serious | Moderate | Low | Low | Low | Moderate | Moderate | Serious |
| Jonjev 2004 | Serious | Low | Low | Low | Low | Moderate | Serious | Serious |
| Sirivella 2005 | Serious | Low | Low | Low | Low | Moderate | Serious | Serious |
| Schwann 2007 | Serious | Low | Low | Low | Low | Moderate | Moderate | Serious |
| Akgun 2008 | Serious | Low | Low | Low | Serious | Moderate | Serious | Serious |
| Marzban 2008 | Serious | Low | Low | Low | Moderate | Moderate | Low | Serious |
| Fang 2022 | Moderate | Moderate | Low | Low | Low | Low | Low | Moderate |

Each domain was rated as Low, Moderate, Serious, Critical, or No Information (NI). Overall judgement reflects the highest-risk domain unless specifically justified otherwise.

**Supplementary Table S3.** Operative technique, reconstruction and conduit, target-vessel specific reporting, and post-operative antithrombotic regimen in the nine included studies.

| **Study** | **CE technique** | **Reconstruction & conduit to CE target** | **Single-vessel CE territory reported** | **Minimum vessel diameter** | **Planned vs intra-operative** | **Post-operative antithrombotic regimen** |
| --- | --- | --- | --- | --- | --- | --- |
| Brenowitz 1988 | Both: RCA often single short arteriotomy; diffuse LAD via long (10–15 cm) open arteriotomy | Saphenous vein split as onlay over LAD arteriotomy; separate vein graft/patch for second distal opening | Not stratified (RCA amenable to single arteriotomy noted qualitatively) | - | - | Low-molecular-weight dextran; antiplatelet use noted (no early-patency effect) |
| Minale 1989 | Both: closed for RCA; long open incision for LAD | Vein-graft onlay over long LAD incision | Not stratified | - | - | Heparin from 6 h → dicoumarine (from POD2) × 6 mo + aspirin (500 mg IV × 2 d, then 100 mg/d) |
| Christenson 1995 | Extensive open LAD endarterectomy (entire cohort) | SVG vs IMA vs IMA + venous patch; IMA-conduit mortality 5.0% vs 9.4% overall | LAD (study is LAD-specific) | - | - | Not specified in detail |
| Jonjev 2004 | Closed / manual traction (all cases); segments 3.5–15 cm | Most anastomoses closed without patch; skeletonised LIMA to LAD/1st diagonal, SVG elsewhere | Not stratified | - | - | Heparin from 24 h → coumadin after clotting-time normalisation (prostacyclin × 24 h = trial arm) |
| Sirivella 2005 | Limited traction (10–12 mm, ~85%); converted to open when distal plaque irregular (~15%) | Conduit (arterial or venous) direct to endarterectomy site; IMA patch-graft for long LAD arteriotomies | Not stratified (OM counter-incision technique described) | ≥1.5 mm outer | - | Antiplatelet (mostly aspirin or clopidogrel) started post-op in all |
| Schwann 2007 | Mills technique (open and closed) | Dedicated conduit study: vein-graft failure to CE target 55% vs 35% non-CE (p=0.05); arterial 13% vs 15% (p=0.88) | Not stratified | ≥1.5 mm external (lumen <1 mm excluded) | - | Not specified as a protocol |
| Akgun 2008 | Both, quantified: open 123 (15.9%) / closed 648 (84.1%) | LIMA to LAD in most closed cases; SVG preferred for open-LAD and calcified small vessels | Not stratified (771 CEs; LIMA-LAD in 128) | - | Decision made intra-operatively after arteriotomy | Low-molecular-weight heparin then warfarin, in addition to aspirin |
| Marzban 2008 | Closed (Watson-Cheyne dissector); arteriotomy ~15 mm, extended distally as needed | Conduit anastomosed end-to-side in all cases | Death-associated combinations reported (LAD+RCA, RCA+diagonal); single territory not stratified | - | Decision made intra-operatively | Heparin reversed at end; restarted 6 h post-op if drainage <100 mL/h; warfarin × 2–3 mo (INR 2.5–3.5) |
| Fang 2022 | Off-pump closed-CE default; open-CE + vein-patch angioplasty if distal plaque incomplete | LIMA always to LAD; sequential SV; bilateral IMA “Y” graft in selected cases | LAD 65 (26.4%) / RCA 134 (54.5%) / multi 47 (19.1%); adjusted MI multi vs LAD-CE OR 9.04 | Target ≥1.5 mm (main lumen <1 mm excluded) | Intra-operative inspection criteria listed | UFH (ACT>180) to extubation; LMWH bridge × 3 d; DAPT × 1 y; single antiplatelet lifelong |

CE, coronary endarterectomy; DAPT, dual antiplatelet therapy; IMA/LIMA, left internal mammary artery; LAD, left anterior descending; LMWH, low-molecular-weight heparin; NR, not reported; OM, obtuse marginal; POD, postoperative day; RCA, right coronary artery; SVG, saphenous vein graft; UFH, unfractionated heparin.

**Supplementary Figure S1.** Bubble plots of meta-regression for each covariate. (A) Triple-vessel disease; (B) Mean LVEF; (C) Diabetes mellitus; (D) On-pump; (E) Publication year; (F) Era.

**Supplementary Figure S2.** Funnel plot for the primary outcome (30-day or in-hospital mortality).
